# A Photoelectrochemical Study of Bioinspired 2-Styryl-1-Benzopyrylium Cations on TiO_2_ Nanoparticle Layer for Application in Dye-Sensitized Solar Cells

**DOI:** 10.3390/ma12244060

**Published:** 2019-12-05

**Authors:** Giuseppe Calogero, Ilaria Citro, Gioacchino Calandra Sebastianella, Gaetano Di Marco, Ana Marta Diniz, A. Jorge Parola, Fernando Pina

**Affiliations:** 1 CNR-IPCF, Viale Ferdinando Stagno d’Alcontres 37, 98158 Messina, Italy; ilariacitro@libero.it (I.C.); Gioakkino.3@live.it (G.C.S.); dimarco@ipcf.cnr.it (G.D.M.); 2 Department of Biomedical, Metabolic and Neural Sciences, University Modena e Reggio Emilia, Via Campi 287, 41121 Modena, Italy; 3 Health Technology College of Lisbon (ESTeSL)—Polytechnic Institute of Lisbon, 1990-096 Lisbon, Portugal; ana.diniz@estesl.ipl.pt; 4 LAQV-REQUIMTE, Departamento de Química, Universidade NOVA de Lisboa, 2829-516 Monte de Caparica, Portugal; fp@fct.unl.pt

**Keywords:** anchoring functional group, dye-sensitized solar cells, self-assembly materials nanocrystalline layers, molecular synthesis, benzopyrylium salts, theoretical calculations

## Abstract

In the present work, five 2-styryl-1-benzopyrylium salts and their relative self-assembly processes towards TiO_2_ nanocrystalline layers were evaluated as photosensitizers in dye-sensitized solar cells (DSSCs). Integration of these 2-styryl-1-benzopyrylium salts with the semiconductor allow for the performance of highly specific functions suitable for smart applications in material science. Spectroscopic and photoelectrochemical measurements conducted on these five bio-inspired dyes, in solution and upon adsorption onto titanium dioxide films, allowed detailed discussion of the anchoring ability of the different donor groups decorating the 2-styryl-1-benzopyrylium core and have demonstrated their ability as photosensitizers. Our results suggest that the introduction of a dimethylamino group in position 4′ of the 2-styryl-1-benzopyrylium skeleton can alter the conjugation of the molecule leading to larger absorption in the visible region and a stronger electron injection of the dye into the conduction band of TiO_2_. Moreover, our experimental data have been supported by theoretical calculations with the aim to study the energy of the excited states of the five compounds. In this specific case, the simulations reported contributed to better describe the properties of the compounds used and to help create the necessary basis for the design of new and targeted bio-inspired molecules.

## 1. Introduction

A complex system is made by individual parts affected by near interactions, of short distance, which cause modifications in the whole system. Scientists can detect these local modifications but cannot predict as the system evolves in the future as a whole. As Edgar Morin (a French Philosopher) says, “in complex systems unpredictability and paradox are always present and some things will remain unknown.” The greater is the quantity and the variety of relations between the elements of a system, the greater its complexity, on condition that the relations between the elements are non-linear. The individual elements determine the overall behavior of the systems and provides them with properties that can be completely unrelated to the individual elements.

This peculiarity is known as emergent behavior, i.e., starting from the interactions between the individual parts of the system a “general behavior” emerges not foreseen by the study of the single components. Examples of these interactions are reported in some scientific articles on self-assembly and bio-architectures are reported by some researchers [1,2,3,4]. Dye-sensitized solar cells are multiparametric and relatively complex systems that work only upon suitable tuning of the properties of the various components of the system (dye, electrolyte, semiconductor). While it is difficult to target a single parameter that controls DSSC’s efficiency, the dye is certainly a critical component and has been subject of, e.g., data mining analysis trying to predict chemical structures for high-performance dyes [5,6].

**Figure 1:**
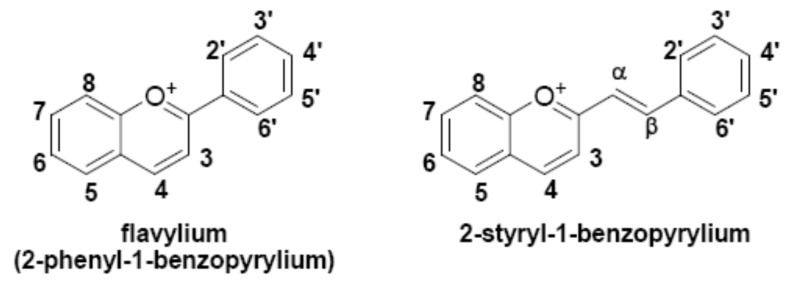
Structure of 2-phenyl-1-benzopyrylium compared with 2-styryl-1-benzopyrylium.

**Figure 2:**
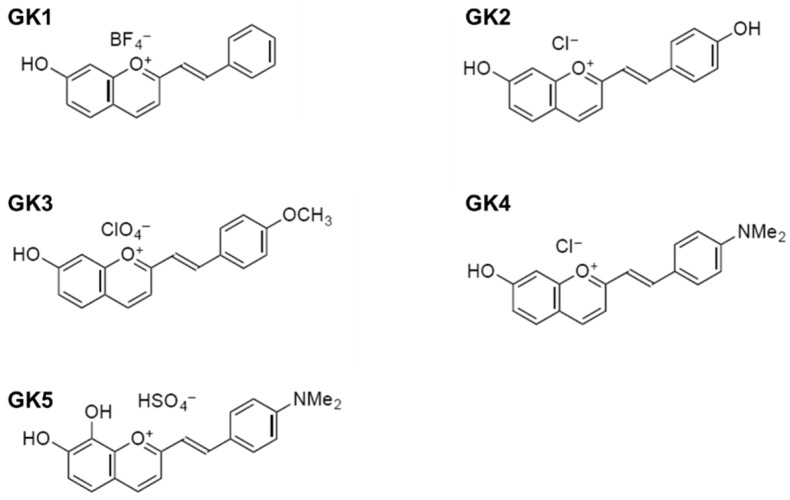
Molecular structures of the five investigated compounds: 7-hydroxy-2-styryl-1-benzopyrylium (GK1), 7-hydroxy-2-(4′-hydroxystyryl)-1-benzopyrylium (GK2), 7-hydroxy-2-(4′-methoxystyryl)-1-benzopyrylium (GK3), 7-hydroxy-2-(4′-dimethylaminostyryl)-1-benzopyrylium (GK4), and 7,8-dihydroxy-2-(4′-dimethylaminostyryl)-1-benzopyrylium (GK5).

In this study, we investigate 2-styryl-1-benzopyrylium salts as dyes for DSSC. These bioinspired compounds are part of the wider family of flavylium dyes, responsible for most of the reds, violets and blues in nature. The color and physico-chemical properties of flavylium dyes [7] as well their application in DSSCs, both as natural extracts [8,9] and as synthetic dyes [10,11,12,13], have been widely studied. Compared to the parent 2-phenyl-1-benzopyrylium (flavylium) structure, the molecular skeleton of 2-styryl-1-benzopyrylium presents an additional double bond connecting the benzopyrylium and the phenyl moieties (see Figure 1). These extended conjugation of the aromatic system leads to larger absorption in the red region of the visible spectrum [14,15,16]. These compounds were initially exploited as food additives in order to confer new color shades [17]. As stronger absorbers in the visible region, they are also prone to be investigated as dyes in DSSC. Figure 2 depicts the five 2-styryl-1-benzopyrylium cations studied in this work. Compounds 7-hydroxy-2-styryl-1-benzopyrylium (GK1), 7-hydroxy-2-(4′-hydroxystyryl)-1-benzopyrylium (GK2), and 7-hydroxy-2-(4′-dimethylaminostyryl)-1-benzopyrylium (GK4) were known form previous studies [14,15], while compounds 7-hydroxy-2-(4′-methoxystyryl)-1-benzopyrylium (GK3) and 7,8-dihydroxy-2-(4′-dimethylaminostyryl)-1-benzopyrylium (GK5) are new compounds. Compounds GK1 to GK4 differ in the substituent in position 4′, while compound GK5 has a catechol group in ring A, allowing to understand the effect of the insertion of the second ortho hydroxyl group in compound GK4.

## 2. Materials and Methods

All chemicals and the other solvents used in this study were used as furnished by chemical companies.

### 2.1. Synthesis

NMR spectra were run on a Bruker AMX 400 instrument operating at 400.13 MHz (^1^H) or 100.00 MHz (^13^C). MALDI MS spectra were acquired on an Applied Biosystems Voyager-DE^TM^ PRO spectrometer.

Styrylflavylium salts 7-hydroxy-2-styryl-1-benzopyrylium tetrafluoroborate (GK1), 7-hydroxy-2-(4-hydroxystyryl)-1-benzopyrylium chloride (GK2), and 7-hydroxy-2-(4-dimethylaminostyryl)-1-benzopyrylium chloride (GK4) were available from previous studies [14,15]. Compounds 7-hydroxy-2-(4-methoxystyryl)-1-benzopyrylium perchlorate (GK3) and 7,8-dihydroxy-2-(4-dimethylaminostyryl)-1-benzopyrylium hydrogensulphate (GK5) were prepared from condensation of the respective salicylaldehydes and styrylmethylketones, according to published methodology [7].

Synthesis of 7-hydroxy-2-(4-methoxyphenyl)-1-benzopyrylium perchlorate (GK3) - 2,4-Dihydroxybenzaldehyde (0.66 g, 4.8 mmol), and *p*-methoxystyrylmethylketone (0.84 g, 4.8 mmol) were dissolved in 5 mL of acetic acid and 1 mL of HBF_4_. 5 mL of acetic anhydride were then added dropwise (ca. 15 min). The temperature was monitored during the addition and it rose until 75 °C and then decreased back to room temperature (22 °C). The reaction mixture was stirred overnight. By the following day, the precipitated solid was filtered off, washed with water, and then carefully with diethyl ether and dried. It was then recrystalized from ethanol / aqueous HClO_4_ (1.17 g, 3.2 mmol). Yield: 66.9%. ^1^H NMR (DCl /CD_3_OD, pD ≈ 1.0, 400.13 MHz) δ (ppm): 8.99 (1H, d, ^3^*J* = 8.4 Hz); 8.52 (1H, d, ^3^*J* = 15.4 Hz); 8.12 (1H, d, ^3^*J* = 9.1 Hz); 7.95 (2H, d, ^3^*J* = 8.0 Hz); 7.88 (1H, d, ^3^*J* = 8.3 Hz); 7.51 (1H, d, ^3^*J* = 15.4 Hz); 7.49 (1H, s); 7.41 (1H, d, ^3^*J* = 9.1 Hz); 7.12 (2H, d, ^3^*J* = 8.0 Hz); 3.94 (3H, s). ^13^C NMR (CD_3_OD/DCl, pD ≈ 1.0, 100.00 MHz) δ (ppm): 172.03; 168.91; 164.61; 159.01; 152.47; 150.20; 132.56; 132.51; 127.32; 120.81; 119.01; 115.49; 114.91; 102.28; 55.00. MALDI TOF MS (+) m/z (%): 278.09 [M]^+^ (100). calcd. for C_18_H_15_O_3_^+^: 279.10 (100%).

Synthesis of 7,8-dihydroxy-2-(4-dimethylaminophenyl)-1-benzopyrylium hydrogensulphate (GK5) - 2,3,4-Trihydroxybenzaldehyde (0.46 g, 3 mmol) and *p*-dimethylaminostyrylmethylketone (0.57 g, 3 mmol) were dissolved in 8 mL of acetic acid and 2 mL of conc. H2SO4. The reaction mixture was stirred overnight. By the following day, ethyl acetate was added and a dark solid precipitated, that was filtered off and carefully washed with ethyl acetate and dried yielding 1.12 g of product (2.75 mmol, 91.6%). ^1^H NMR (DCl /CD3OD, pD ≈ 1.0, 400.13 MHz) δ (ppm): 8.84 (1H, d, ^3^*J* = 8.6 Hz); 8.55 (1H, d, ^3^*J* = 15.6 Hz); 7.99 (2H, d, ^3^*J* = 8.9 Hz); 7.79 (1H, d, ^3^*J* = 8.6 Hz); 7.63 (1H, d, ^3^*J* = 8.8 Hz); 7.52 (1H, d, ^3^*J* = 15.6 Hz); 7.48 (2H, d, ^3^*J* = 8.9 Hz); 7.38 (1H, d, ^3^*J* = 8.8 Hz); 3.29 (6H, s). ^13^C NMR (CD_3_OD/DCl, pD ≈ 1.0, 100.00 MHz) δ (ppm): 171.89; 157.75; 153.16; 151.07; 150.66; 149.71; 147.48; 134.41; 134.02 (2C); 124.02; 120.86; 120.60; 119.5 (2C); 116.86; 44.53. MS MALDI TOF (+) m/z (%): 308.07 [M]^+^ (100%); calcd. for C_19_H_18_NO_3_^+^: 308.13 (100%).

### 2.2. Devices Fabrication

The details of fabrication of the anodes and UV vis spectra are reported somewhere else [11]. The active area of DSSC was 0.196 cm^2^ with a thickness of ~18 μm (see Figure S1) while the anode employed for the absorption spectra were larger (TiO_2_ active area 1 cm^2^) and thinner (TiO_2_ layer thickness 4 μm).

Avoiding moisture absorption, all the prepared anodes were stores in oven at about 80 °C until their employment.

Sensitizer solutions were prepared by dissolving the compounds in water, ethanol, acidified water, or acidified ethanol. Aqueous solutions were acidified with HCl until the pH was in the range 1 < pH < 2, as measured by pHmeter (HANNA Instrument) calibrated with standard buffer solutions of 10.01, 4.01, and 7.00 buffer solution (by HANNA Instruments). Ethanolic solutions were acidified to pH = 1.5 by adding aqueous HCl to absolute ethanol. Obtained solutions were stored in a refrigerator at +5 °C and protected from light exposition for several months. The photoanodes were prepared by soaking the anodes overnight in the dye solutions, at room temperature in a dark box. The excess dye was removed by rinsing the photoanodes with the respective solvents (water or ethanol acidified). Finally, these photoanodes were dried at 80 °C for 30 min.

Each counter-electrode was a conductive glass (rectangular area: 4 cm^2^) predrilled. The catalytic layer was deposited on the conductive face of the glass employing a H_2_PtCl_6_ isopropanolic solution (5 mM) which was sintered at 500 °C for half an hour. The electrodes were assembled into a sandwich-type arrangement, employing a thermo-pneumatic press to seal the DSSC with a polymeric frame. 

The electrolyte (AS8*) were prepared as described in a previous paper [11]. 

### 2.3. Instrument Setup

A Bruker DektakXT profilometer (Bruker Italia s.r.l., Milano, Italy) was employed to measure the thickness of the TiO_2_ layer, while a Perkin Elmer L20 UV-Vis (Perkin Elmer Italia Spa, Milano, Italy) was employed to record absorption spectra. I-V curves were measured by a digital Keithley 236 multimeter (Tektronics Company, Padova, Italy) connected to a PC and controlled by an open source software. A LOT-Oriel solar simulator (Model LS0100-1000, 300 W Xe Arc lamp powered by LSN251 power supply equipped with AM 1.5 filter calibrated by a LP PYRA 02 AC pyranometer). The short circuits current (*Isc*), the open-circuit voltage (*Voc*), the fill factor (*FF*) and the Power Conversion Efficiency (*η*) were calculated from the I-V data. *FF* was calculated by the following equation:

(1) $$FF=\frac{I_{max}\times V_{max}}{I_{SC}\times V_{OC}}$$

where the product of *V_max_* and *I_max_* are voltage maximum and current maximum of the DSSC. The Power Conversion Efficiency (*η*) was determined by the equation:

(2) $$\eta =\frac{I_{SC}\times V_{OC}\times FF}{P_{in}}$$

where *P_in_* was the power of incident light. Usually, a black background was employed at the back of the investigated DSSCs.

### 2.4. Computational Methods

Computational calculations of the primary structures of the five cations were elaborated with the software Gauss View 4.1 (4.1.2, Gaussian Inc., Wallingford, USA). The molecules’ ground state geometries, dipole moments, and frontier orbital plots of HOMO (Highest Occupied Molecular Orbital) and LUMO (Lowest Unoccupied Molecular Orbital) were calculated and drawn by Gaussian 03 software processed by a Fujitsu workstation. The optimization of the ground state geometries of the five styryl-1-benzopyrylium cations was performed using the calculation method of the density functional theory (DFT) with functional B3LYP and the base set 6-31 + G (d) [18,19]. The elaboration of the electron density, of the HOMO and LUMO of the cations studied, the energies of the electronic excitations and the oxidation potentials were calculated using the functional theory of time-dependent density (TDDFT) with the same functional (B3LYP) functional and same basis as mentioned above [20]. The calculations were processed without considering the contribution due to the presence of solvents [21].

## 3. Results and Discussions

### 3.1. Orbital Energy and DFT Calculations

Table 1 shows electron densities of 2-styryl-1-benzopyrylium cations calculated by the Gaussian software 03 [22]. Careful analysis of the HOMO–LUMO difference in electron density for each compound shows that in the case of compounds GK1 and GK2 the electron density tends to move to the phenyl group while an inverse tendency is observed for GK3 due to the presence of the methoxy (-OCH_3_) group that is a better electron-donor than OH. Also, in the case of GK4 and GK5, the electron donor ability of the dimethylamino group in position 4′ leads to a displacement of electron density toward the benzopyrylium moiety.

**Figure 3:**
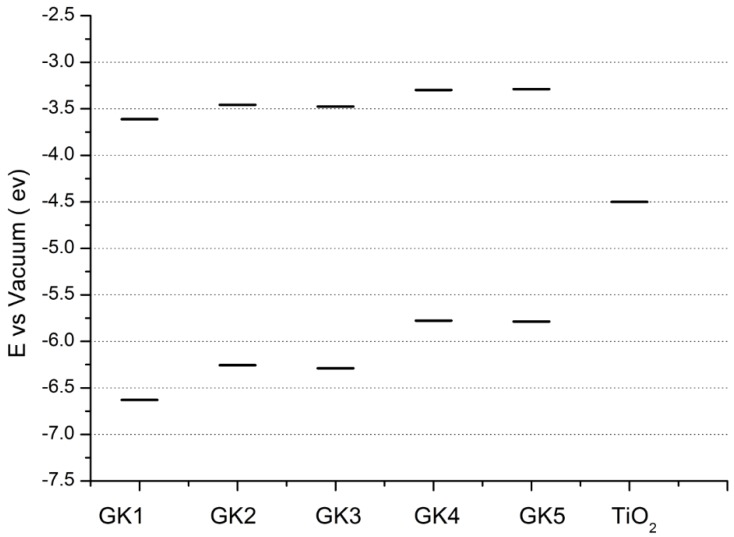
Energy levels of the five investigated dyes (G1, GK2, GK3, GK4, GK5) calculated by Gaussian 03.

In Figure 3 and in the Table 2, calculated energies of the HOMO and LUMO are presented. One can immediately see that all compounds have an energy level of the excited state higher than that of the conduction band of TiO_2_. This means that from the point of view of energy, all dyes would be able to inject an electron from their excited LUMO level to the conduction band of the titanium oxide semiconductor. However, this is not enough to get that to happen, as the main condition which must be respected is for the dye to act as a sensitizer that is anchored firmly to the semiconductor substrate, sensitizing precisely the photoanode. As we have seen previously, not all the studied dyes are capable of anchoring because of the reasons explained previously.

### 3.2. Optical Properties and Adsorption on TiO_2_

**Figure 4:**
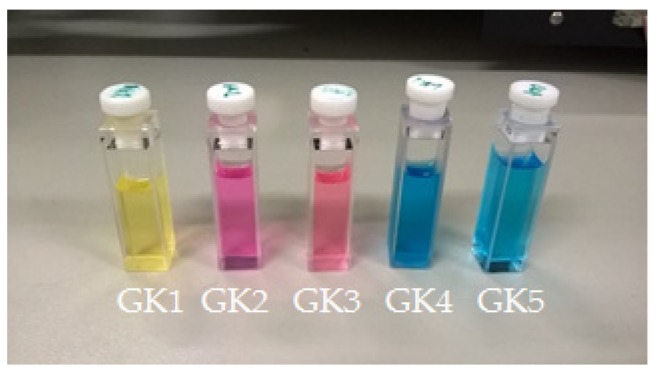
Palette of the water solution of the five investigated dyes (GK1-GK5).

**Figure 5:**
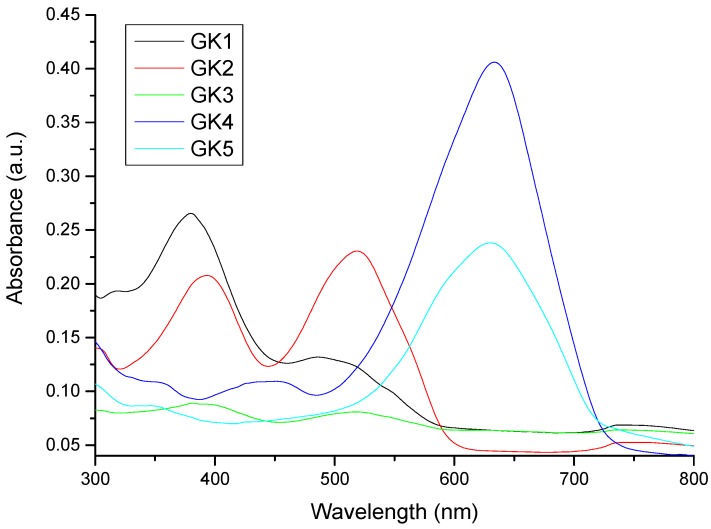
Absorption spectra of the five investigated compounds dissolved in water.

Compounds GK1-GK5 are characterized by reasonable solubility in water and the respective colors are presented in Figure 4 with absorption spectra shown in Figure 5. All dye solutions show to absorb visible light. In particular, compounds GK1-GK3 are characterized by the presence of two absorption bands. In compound GK1, these bands show up at 380 nm and 478 nm, while in both GK2 and GK3, these bands are centered at 395 and 520 nm. The redshifts observed in compounds GK2 and GK3 relative to compound GK1 arise from the presence of hydroxyl and methoxy group, respectively. These electron donor groups extend the conjugation and accentuate the charge transfer character of the transition, lowering the energy of the HOMO–LUMO transition in accordance with trend in transition energies predicted from calculations (cf. Table 2).

**Figure 6:**
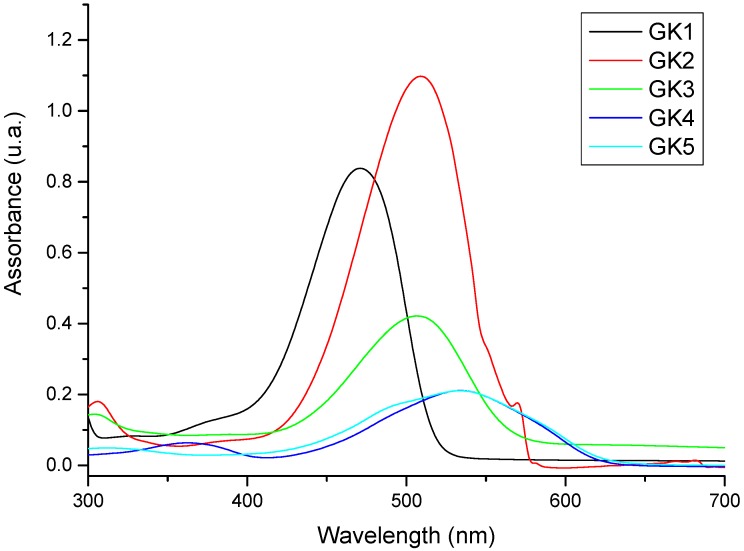
Absorption spectra of the five investigated compounds dissolved in water under acid conditions.

Compounds GK4 and GK5 present a main transition, centered at 635 nm in both compounds. This band is markedly redshifted relative to the lowest energy transitions in compounds GK1-GK3, manifesting the stronger electron–donor ability of the dimethylamino group compared to OMe (GK23), OH (GK2) and H (GK1). The introduction of an additional OH group in GK4 transforming it into GK5 acts only on the intensity of the absorption, but not on the position of the maximum peaks. From this first study, all five compounds would be ideal candidates for use as sensitizers in Grätzel cells, since they all absorb light in the visible region typical of solar radiation. It is known that flavylium salts may adsorb better to TiO_2_ from acidic than from neutral solutions [10,11,12,13]. Further study on the solubility and absorption properties was done by preparing aqueous acidic solutions of the compounds (Figure 6) as well as ethanolic (Figure 8) and acidified ethanolic solutions (Figure 9).

The absorption spectra in aqueous acidic solution (Figure 6) differ substantially from the spectra in water (Figure 4). All compounds are now characterized by a main absorption band while in neutral solution, compounds GK1-GK3 presented two bands. Flavylium as well as styrylflavylium compounds are known to exist as chemical reaction networks where different chemical species are connected through several equilibria and the mole fraction distribution of species depends on the pH of the solution [7].

**Figure 7:**
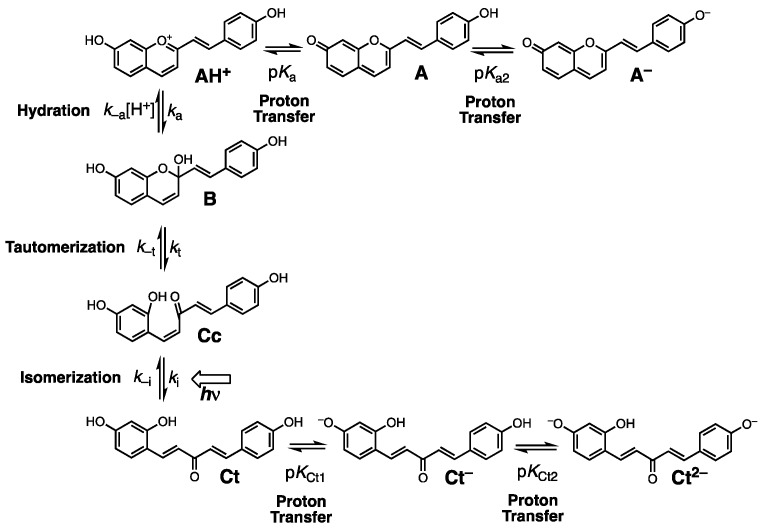
Network of reactions in H_2_O of GK2.

Figure 7 depicts this equilibria network for compound GK2 [14,15]. The styrylflavylium cation (AH^+^) is the stable species at acidic pH values. When the pH is increased, AH^+^ undergoes proton transfer and hydration reactions to form quinoidal base (A), hemiketal (B), and chalcones (Cc and Ct), that are the predominant species at moderately acidic and neutral pH values. At basic pH values, if phenol groups are present, these species deprotonate further to give anionic species (e.g., A^−^, Cc^−^, Ct^−^), while at strongly acidic conditions, if amino groups are present, species protonated at the nitrogen are formed (AH_2_^2+^, B^+^, Cc^+^, Ct^+^; not shown).

**Figure 8:**
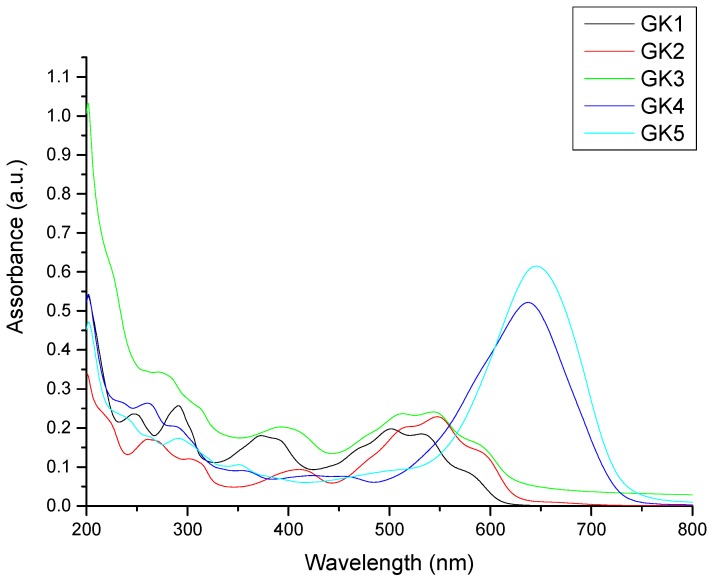
Absorption spectra of the five investigated compounds dissolved in ethanol.

**Figure 9:**
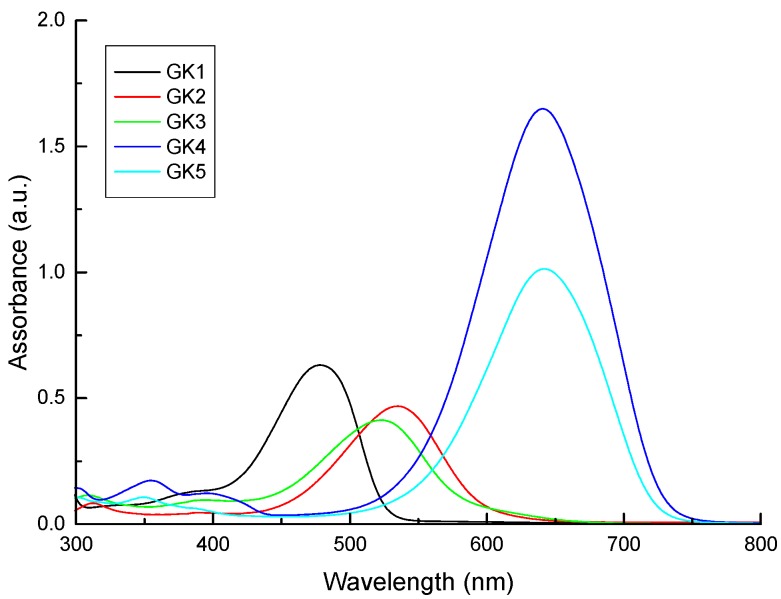
Absorption spectra of the five investigated compounds dissolved in acidified ethanol.

Absorption spectra both in ethanol and in acidified ethanol (Figure 8 and Figure 9) exhibit a general redshift of the absorption maxima when compared to spectra in aqueous solution. This is due to the known negative solvatochromism of flavylium cations, i.e., their visible absorption band is shifted towards longer wavelengths with decreasing polarity of the solvent [23,24]. Negative solvatochromism is due to a better stabilization of the flavylium electronic ground state relatively to its less polar excited state when increasing solvent polarity. In ethanol (Figure 8), the lowest energy transition in GK1, GK2, and GK3 show vibrational resolution which is a spectral fingerprint for the presence of the quinoidal base species [7] as predominant species, together with hemiketal and chalcones that account for the higher energy transitions. Similarly to what is observed in aqueous solution, acidification of the ethanolic solutions gives to spectra with one main absorption band corresponding to the AH^+^ species. In ethanol, however, the protonated species observed in acidic water (AH_2_^2+^, B^+^, Cc^+^, Ct^+^) are more difficult to solvate and the bands from GK4 and GK5 are quite similar in ethanol and in acidic ethanol and are assigned to the styrylflavylium.

**Figure 10:**
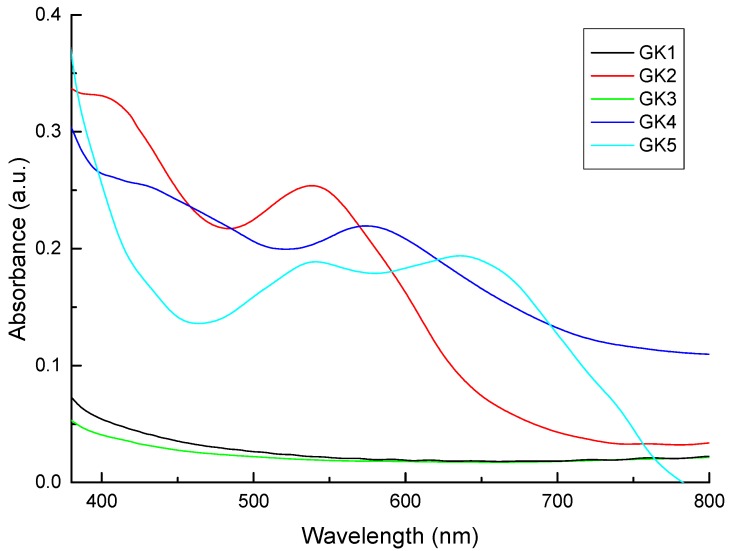
Absorption spectra of the photoanodes obtained by soaking the anodes in aqueous solutions of the of five investigated dyes.

**Figure 11:**
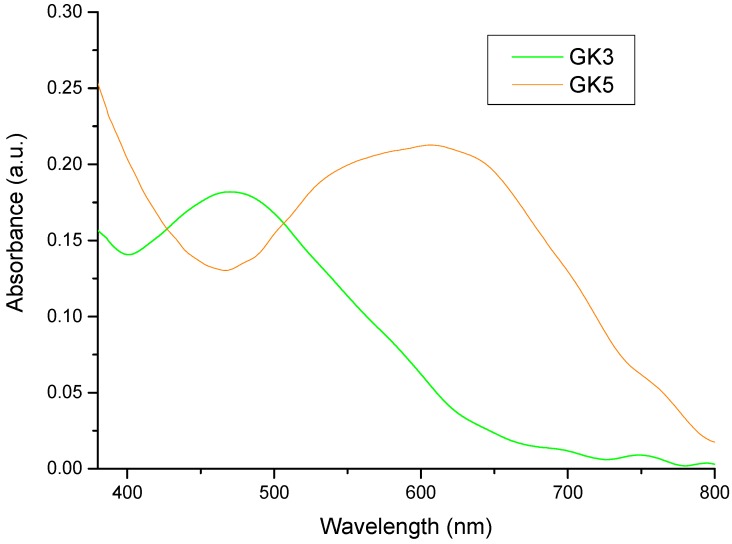
Absorption spectra of the photoanodes GK3 and GK5 obtained by soaking the anodes in acidified ethanolic solutions. GK1, GK2, GK4 were not reported in this Figure. because of too low absorption, but their pictures are reported in Figure S2 (see Supporting Information).

Titanium dioxide photoanodes were soaked in four different solutions (water, acidified water, ethanol, acidified ethanol) for each of the five dyes; 20 solutions overall. The absorption spectra of the photoanodes that shown in Figure 10 and Figure 11 and the absorption results summarized in Table 3. Compound GK1 did not adsorb on TiO_2_ films from any of the four solutions, while compound GK5 absorbed well from all of them. The other compounds adsorbed either from water or from acidified ethanol and these two solvents were those overall more relevant chosen to present the results.

The fact that styrylflavylium GK1 does not adsorb from any of the solvents shows that OH group in position 7 has poor ability to form a bond with titanium. Formation of such a bond would involve deprotonation of the phenol with formation of a zwitterion. The negative charge developing on the oxygen atom delocalizes to neutralize the positive charge in the pyrylium ring, giving the most stable resonance structure due to the neutral quinoidal base (structure A in Figure 7). This decreases the electron donation ability of the 7-OH group and prevents dye adsorption to TiO_2_. A similar situation was observed for 4′-hydroxyflavylium in a previous paper, with poor adsorption and very low efficiency of the respective DSSC [10]. Whenever OH groups alone are present in resonant positions of the flavylium or styrylflavylium core, poor dye performance in DSSC is to be expected. Styrylflavylium GK2 has a second hydroxyl group in position 4′ and is able to adsorb to TiO_2_. It may adsorb through the 7-OH group receiving electrons from the oxygen donor in position 7 or the opposite may happen: adsorption through 7-OH with electrons conveyed from the oxygen in position 4′ along the π bridge defined by the styryl skeleton in its quinonoid form (as in structures A and A^–^ in Figure 7). In help of the feasibility of this second hypothesis comes the fact that compound GK3 adsorbs to TiO_2_ from one of the solvents and it can only bind to Ti(IV) through the oxygen atom in position 7.

Compounds GK4 and GK5 both adsorb to TiO_2_ and have a dimethylamino group in position 4′. This electron donor group delocalizes the pyrylium charge to the styryl moiety allowing adsorption of GK4 through the oxygen in position 7. Alternatively, adsorption can take place through the amino group. The absorption maximum of this photoanode occurs at 574 nm (Figure 10) and is blue-shifted relative to the dye in solution (636 nm), suggesting involvement of the nitrogen lone pair in Ti(IV) binding. For styrylflavylium GK5 there are also two possibilities: The first, in analogy to what seems to be happening in compound GK4, adsorption occurs via the nitrogen atom; the second involves Ti(IV) complexation through the catechol moiety. Catechol groups have proved to be particularly efficient in Ti(IV) binding in natural [9,25] as well as in synthetic flavylium dyes [10,11,12,13]. In support of this reasoning, absorption spectra of the two GK5 photoanodes, either soaked in water or in acidified ethanol, are not blue-shifted in comparison with the dye in solution, suggesting that the nitrogen is poorly involved in the adsorption process that would be guaranteed by the catechol. Nevertheless, contribution of the amino group in the adsorption to TiO_2_ cannot be excluded if we consider that the catechol group may be involved in hydrogen bonding (see below).

### 3.3. Photoelectrochemical Characterization of DSSCs

**Figure 12:**
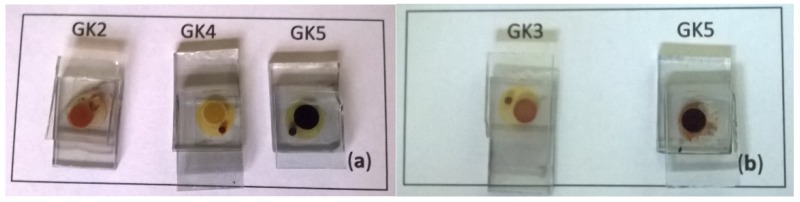
Assembled dye-sensitized solar cells (DSSC) based on compounds (**a**) GK2 (H_2_O), GK4 (H_2_O), GK5 (H_2_O); (**b**) GK3 (C_2_H_5_OH/H^+^), GK5 (C_2_H_5_OH/H^+^).

The I-V curves and the photoelectrochemical parameters of some selected assembled DSSCs (see Figure 12) are presented in Table 4. DSSCs based on compounds GK4 and GK5 exhibit the highest currents, more than twice that observed for GK2. These results reflect the effectiveness of the dimethylamino group in injecting electrons on TiO_2_ when compared to hydroxyl or methoxy groups.

Compound GK1, as seen above, does not adsorb onto TiO_2_ photoanode. In fact, looking at the molecular structures of these dyes, the OH group in position 7 hardly binds TiO_2_. Styrylflavylium GK2, possessing another OH group in position 4′, binds to TiO_2_ through the latter (or through the 7-OH) and injects an electron into the conduction band of TiO_2_. As shown in Table 4, this takes place in aqueous solution. The energy conversion efficiency for GK2 is still very low, despite being anchored and the same is observed for compound GK3. The corresponding currents are as well very low, testifying that the OH functional groups in these positions are poor injector groups. In compound GK4, anchoring to the TiO_2_ occurs via the amino group. We see immediately that the efficiency and all other parameters (Jsc, Voc, FF) are much higher than in previous compounds, suggesting that the direct bond formed between the amino group and titanium efficiently allows the passage of electrons between the ligand and the metal. To better understand the difference between the two types of anchoring, we can analyze the behavior of compound GK5 that contains both functional groups, and where the presence of 7-OH is reinforced by a second hydroxyl in position 8. In the case in which GK5 comes from an acidified aqueous solution, we have the maximum current and maximum yield. Apparently, this is due to protonation in the amino group favoring the attachment of catechol to TiO_2_. In reality, this may not be true because the catechol in water maintains the ability to form stable hydrogen bonds. Also, the amount of acid present is less than the number of functional groups, part of the amino groups may be free to bind TiO_2_ as in compound GK4; in fact, GK5 shows a good efficiency and a good current in water. When GK5 is instead dissolved in acidified ethanol, protonation of the amino group is more effective and the relative efficiencies and current are slightly lower. When we look at the behavior of the photoanode GK5 finally obtained from aqueous solutions, we see that it is very similar to that obtained in acidified water and in this case the amino is entirely free, while the catechol is engaged in hydrogen bonds with water [5]. A last consideration can be made by looking at the values of Voc in GK4 and GK5 that are the highest except when the GK5 is prepared from the acid solution, in that case is lowered since the doublet of the amino does not change the level of the conduction band of TiO_2_ [6,7].

## 4. Conclusions

In the present work, five 2-styryl-1-benzopyrylium salts were used as photosensitizers in DSSC. Spectroscopic and photoelectrochemical measurements have demonstrated their strong potential in order to be used for photovoltaic applications. The best results were obtained with styrylflavylium GK4, that when subjected to an irradiance of 100 mWcm^−2^, has provided a sunlight–electric current conversion efficiency of 1.27%, a current density Jsc of 5.519 mAcm^−2^, a fill factor of 61%, and a voltage Voc of 389 mV. These results suggest that the introduction of a dimethylamino group in position 4′ of the styrylflavylium skeleton, can alter the conjugation of the molecule by arranging a greater absorption in the visible region and a better injection electronic part of the dye to the conduction band of TiO_2_.

Although UV–Vis absorption and current–voltage data are critical to this study, computational calculations have enriched with useful information. More and more often in the literature experimental data were in fact supported by theoretical calculations or simulations on the one hand, and in this specific case, the simulations reported contributed to better describe the properties of the compounds used and to help create the necessary basis for the design of new and targeted organic photosensitizers. 

## Figures and Tables

**Table 1:** Density functional theory (DFT) images of 2-styryl-1-benzopyrylium calculated by Gaussian 03.

HOMO	LUMO	Dye
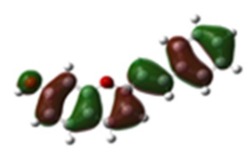	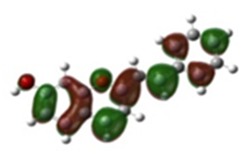	GK1
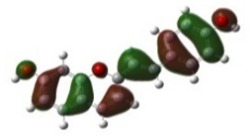	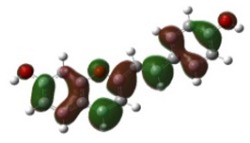	GK2
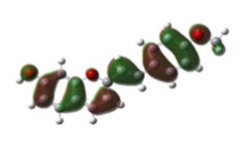	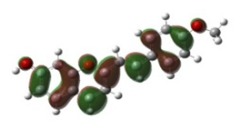	GK3
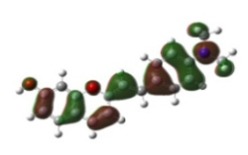	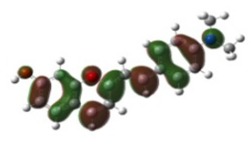	GK4
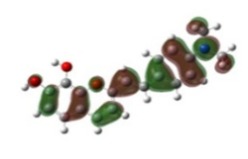	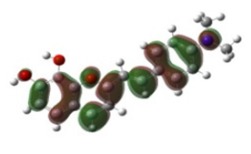	GK5

**Table 2:** Molecular orbital and theoretical absorption maximum wavelength calculated by computational methods for the five investigated cations in ethanol.

Dye	Molecular Orbital (eV)				Absorption Maximum (nm)	
	HOMO-1	HOMO	LUMO	LUMO+1	Theoretical	Experimental
**GK1**	−73,975	−6629	−3611	−21,020	453	478
**GK2**	−71,703	−6256	−34,579	−19,701	477	536
**GK3**	−71,711	−6289	−3476	−19,894	479	523
**GK4**	−69,091	−5777	−3299	−18,460	522	640
**GK5**	−64,378	−5786	−3288	−18,422	521	642

**Table 3:** Absorption wavelength range of the dyes in water and in acidified ethanol and of their corresponding photoanodes.

Dye	H_2_O/nm	Photoanode from H_2_O/nm	EtOH_H^+^/nm	Photoanode from EtOH_H^+^/nm
GK1	380–486	No sensitization	312–Sh_383–478	No sensitization *
GK2	394–519	538	536	Colorless *
GK3	582–527	Colorless	Sh_394–523	474
GK4	Sh_350 451–633	Sh_342 574	355–396–640	Colorless *
GK5	Sh_346 640	541–636	Sh_382–642	607

* See pictures Figure S1 in supporting information.

**Table 4:** Photovoltaic parameters (current density, J_SC_; open circuit voltage, V_OC_; fill factor, FF; and efficiency, η) and performances for the solar cells sensitized by water solution of GK2, GK4, and GK5 and ethanolic acidic solution of GK3 and GK5.

Dyes	Solvent Used for Soaking	Jsc (mA/cm^2^)	Voc (mV)	FF	η%
**GK2**	H_2_O	2.240	246	0.38	0.22
**GK3**	C_2_H_5_OH/H^+^	1.380	255	0.58	0.21
**GK4**	H_2_O	5.519	389	0.61	1.27
**GK5**	H_2_O	6.408	291	0.54	1.06
**GK5**	C_2_H_5_OH/H^+^	3.100	218	0.54	0.37

## Supplementary Materials

The following are available online at https://www.mdpi.com/1996-1944/12/24/4060/s1, Figure S1: SEM cross-sectional image for the anode, Figure S2:—Photoanodes for absorption spectra sensitized by ethanol acidified solution (pH 1.5).
