# Supplementary material for: A Photoelectrochemical Study of Bioinspired 2-Styryl-1-Benzopyrylium Cations on TiO2 Nanoparticle Layer for Application in Dye-Sensitized Solar Cells
Source: Materials (Basel). 2019 Dec 5;12(24):4060. doi: 10.3390/ma12244060 (PMC6947086; doi:10.3390/ma12244060)
Supplement: Supplementary file 1 [file materials-12-04060-s001.pdf]

materials

Article

A Photoelectrochemical Study of Bioinspired 2-Styryl-1-Benzopyrylium Cations on TiO_2 Nanoparticle Layer for Application in Dye-Sensitized Solar Cells

Giuseppe Calogero [1,*], Ilaria Citro [1], Gioacchino Calandra Sebastianella [1,2], Gaetano Di Marco [1], Ana Marta Diniz [3], A. Jorge Parola [4,*] and Fernando Pina [4]

[1] CNR-IPCF, Viale Ferdinando Stagno d'Alcontres 37, 98158 Messina, Italy; ilariacitro@libero.it (I.C.); Gioakkino.3@live.it (G.C.S.); dimarco@ipcf.cnr.it (G.D.)
[2] Department of Biomedical, Metabolic and Neural Sciences, University Modena e Reggio Emilia, Via Campi 287, 41121 Modena, Italy
[3] Health Technology College of Lisbon (ESTeSL) -- Polytechnic Institute of Lisbon, 1990–096 Lisbon, Portugal; ana.diniz@estesl.ipl.pt
[4] LAQV-REQUIMTE, Departamento de Química, Universidade NOVA de Lisboa, 2829–516 Monte de Caparica, Portugal; fp@fct.unl.pt
* Correspondence: giuseppe.calogero@cnr.it (G.C.); ajp@fct.unl.pt (A.J.P.); Tel.: +39–090–39762247 (G.C.); +351–212948300 (A.J.P.)

Received: 30 October 2019; Accepted: 3 December 2019; Published: date

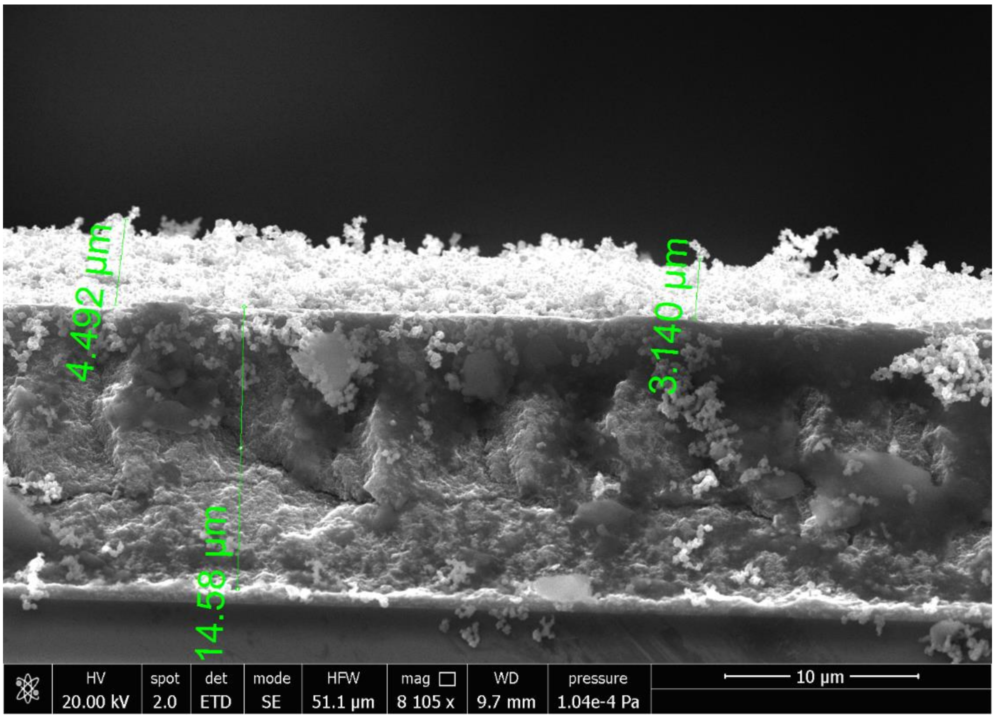

Figure S1. SEM cross-sectional image for the anode

In the picture of Figure S1 are showed the SEM cross sectional image (captured by a SEM QUANTA FEG 450) of the TiO_2 anode used for the DSSC and can be see two zones: the former constituted by the transparent layer of commercial TiO_2 nanoparticles (diameter 10–15 nm) with a thickness of around 14,5 µm, and the latter, on the top, constituted by the scattering layer of commercial TiO_2 nanoparticles (diameter 150–200 nm) with a thickness of around 4 µm, depending of the irregularity due to the large TiO_2 nanoparticles of 150–200 nm.

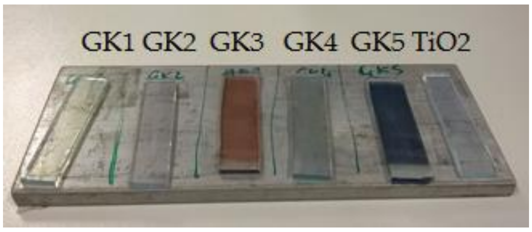

Figure S2. – Photoanodes for absorption spectra sensitized by ethanol acidified solution (pH 1.5)

In Figure S2 are reported the images of the photoanodes for the five dyes and a TiO_2 anode used for UV-Vis spectra, unfortunately for GK1 GK2 and GK4 the sensitization was very low and we cannot reproduce absorption spectra for them because of very low signal and of the background.
